# Predicting commitment in university students: the role of collective trust and self-efficacy

**DOI:** 10.3389/fpsyg.2025.1643129

**Published:** 2025-10-21

**Authors:** Happy Joseph Shayo, Nilo Jayoma Castulo, Frederick Oduro, Arlyne C. Marasigan, Rambo LY, Sarfraz Aslam

**Affiliations:** ^1^Human Capital Management and Administration, Moshi Co-operative University, Moshi, Tanzania; ^2^Department of Educational Leadership and Professional Services, College of Education, Mindanao State University - Tawi-Tawi College of Technology and Oceanography, Bongao, Philippines; ^3^Institute of International and Comparative Education, Beijing Normal University, Beijing, China; ^4^College of Advanced Studies, and Education Policy and Research Development Office, Philippines Normal University, Manila, Philippines; ^5^Faculty of Sociology and Community Development, National University of Battambang, Battambang, Cambodia; ^6^Faculty of Education and Humanities, Unitar International University, Petaling Jaya, Malaysia

**Keywords:** affective commitment, collective trust, higher education, self-efficacy, social identity theory

## Abstract

**Objective:**

This study tested whether collective trust in supervisors predicts university students’ affective commitment and whether student self-efficacy mediates this relationship. Demographic variables (age, sex, grade level, and major) were evaluated as potential moderators.

**Method:**

A cross-sectional, explanatory, non-experimental survey was administered to 968 students at a normal Chinese university. Measures included an adapted collective trust scale, an eight-item general self-efficacy scale, and an affective commitment subscale. Data screening confirmed univariate normality. A two-step covariance-based SEM procedure was conducted in AMOS 24: confirmatory factor analysis established a 17-item measurement model, and the structural model tested the direct and indirect paths. Composite scores were computed for descriptive analysis. Mediation was evaluated with bootstrap resampling (2000), and moderation tests used Hayes’s PROCESS macro for SPSS.

**Results:**

The final measurement model demonstrated an acceptable fit. SEM results indicated that collective trust positively predicted affective commitment and self-efficacy with moderate effect sizes, while self-efficacy positively predicted affective commitment (weak effect); together, these predictors accounted for approximately 49.8% of variance in affective commitment, indicating a strong effect. Bootstrap mediation revealed a significant indirect effect of collective trust on commitment through self-efficacy (partial mediation). Moderation analyses produced partial support: age and grade level moderated the self-efficacy → commitment link, academic major moderated the trust → self-efficacy link, and most other interactions were non-significant.

**Conclusion:**

The findings highlight the joint importance of group-level trust and individual efficacy for institutional attachment and suggest the value of cluster-sensitive interventions. Longitudinal and multi-site research is recommended to confirm causal pathways and boundary conditions.

## 1 Introduction

Higher education institutions are considered as transformation agents (Sporn and Godonoga, 2024), implying that university life is a pivotal experience for aspiring scholars, significantly influencing their careers. Universities play a crucial role in fostering committed students, with commitment being a vital component of the student experience (Cownie, 2019; Cownie and Bradney, 2017). Both negative and positive experiences are instrumental in shaping affective commitment (Arriaga and Agnew, 2001). Commitment represents the drive to remain engaged in a relationship or task and to work diligently. It encourages individuals to forgo personal interests and short-term gains, curbing immediate and undesirable impulses for the relationship’s greater good. Consequently, affective commitment, which reflects students’ emotional desire to connect with their educational institutions, is a significant phenomenon in higher education (Cownie and Bradney, 2017). It is a powerful catalyst for loyalty in higher education (Bowden and Wood, 2011). Since it is constructed from experiences, interaction among students and faculty cannot be exempted, however, studies put more weight on the influence of trusting interpersonal relationships with faculty members (Seth and Bhuyan, 2023; Tormey, 2021). Notably, although students exhibit higher levels of affective commitment to their university compared to their academic instructors, it is the affective commitment to academics that most significantly impacts students’ intentions to speak positively (Cownie, 2020). Therefore, universities through faculty members enhance student experience to foster engagement and success, for student’s commitment, and sense of belongingness boost the institution’s reputation. Additionally, commitment not only depend on external influence, but also internal motive toward achievement of a goal, hence necessitates the existence of self-trust and courage (Bandura, 1977).

The interpersonal relationship between students and faculty members is prominent during supervision and mentorship. It significantly influences the experiences of both educators and learners in higher education and has been associated with learning, classroom management, and student absenteeism (Tormey, 2021). Supervision at Chinese universities revolves around three key aspects: tasks and roles (Lee, 2018; Tahir et al., 2012; Woolderink et al., 2015), relationships (Hemer, 2012), and expectations (Ali and Watson, 2016). A study in China highlighted a collaborative relationship marked by supervised freedom that allows learners to choose their research interests (Wang and Byram, 2019). In addition to scheduled personal meetings with supervisors, some faculty members hold weekly seminars for graduate students where all supervisees under the same supervisor present their research progress in the presence of their supervisors. These seminars foster student commitment, knowledge sharing, social interaction, and presentation skills. On the other hand, the undergraduate students receive mentorship meetings that build a sense of fulfilment, research dedication and self-confidence (Hu and Zhou, 2024). Students at Chinese universities have expressed a sense of “family bond” with their supervisors (Wang and Byram, 2019; Wu, 2006), reflecting an affective commitment among them. However, supervisors view their relationship with students as a teacher-friend dynamic, or “yi shi yi you [亦师亦友]” (Wang and Byram, 2019). Supervisors act as role models in both academic and social contexts, a concept known as “jiao shu yu ren [教书育人],” which signifies their responsibility to educate and cultivate “good character” in learners (Wang and Byram, 2019). Moreover, the supervisors express these connections as informal reverse mentoring for they get opportunity to learn from the students (Hu and Zhou, 2024; Roberts and Seaman, 2018). Regardless of the existing bond between university students and their supervisors, it is still unclear whether the constructed experiences with the faculty and self-confidence affect student sense of belonging and attachment toward their university. This study therefore, explores how university students’ commitment toward their institution can be cultivated through self-confidence and trust in the faculty.

### 1.1 Theoretical development

This study falls into multiple disciplines, such as psychology and sociology, and requires the integration of various theories to understand the examined constructs. We integrated self-determination theory [SDT] (Deci and Ryan, 2012, 2014) and social identity theory (Tajfel and Tunner, 2004) to understand the relationships and effects among the complex constructs of collective trust, self-efficacy, and affective commitment.

#### 1.1.1 Relationship motivation theory (RMT)

Relationship Motivation Theory (RMT) is a mini-theory of SDT that describes relational interactions and suggests that relationships are essential for satisfying the need for relatedness (Deci and Ryan, 2014). Student-supervisor relationship is a mutual interaction that requires trust to facilitate the attainment of educational goals (Yan et al., 2024). Drawing from the nature of supervision and mentorship activities, “supportive supervisory relationships, directing learning to empower students, and an alignment of student-supervisor interests and approaches” stem from mutual trust that makes students feel recognized and capacitated as intellectuals (Roberts and Seaman, 2018, pp. 2–3). These feelings can be explained by the RTM theory of motivation in terms of relations that provide a sense of autonomy. When students are listened to and their work or ideas are accepted by their supervisors, they develop a sense of relatedness to their university, which may lead to affective commitment toward the institution. While commitment, in general, is an individual’s decision to act on what motivates them, this theory contributes to understanding the motivation behind student affective commitment—an emotional attachment to a particular task or person—as a result of interaction with their supervisors. Consequently, examining ways to stimulate and secure students’ affective commitment is essential in higher education (Cownie and Bradney, 2017; Mercurio, 2015), given that the effect of individuals is inconsistent and complex to control (Mercurio, 2015; Meyer and Herscovitch, 2001).

#### 1.1.2 Social identity theory (SIT)

Trust in supervisors is one aspect that may influence self-confidence and commitment, however, the nature of supervision activities in higher education is dominated with group meetings as compared to individual interactions given the high number of students (Hu and Zhou, 2024; Yan et al., 2024). We applied the Social Identity theory that acknowledge individuals defining their sense of self through social groups. Self-identity is a product of social interactions, in which one identifies where one belongs based on the group’s characteristics and cultural norms (Tajfel and Tunner, 2004). Supervisor’s responses during group mentorship may develop self-confidence and sense of belonging (ingroup) to the supervisee, hence promote collective trust (Brower et al., 2009; O’Doherty, 2023) and positive interaction with self and other group members. Collective trust, defined as a collective norm in a higher education institution, may shape learners’ social identity and their beliefs (self-efficacy) (Forsyth et al., 2011). Examining individual interactions and relationships will likely contribute to understanding their behavior and beliefs. Moreover, the feeling of belonging can be the outcome of a social interaction culture (collective trust) or a psychological state of self (self-efficacy) built on social obligations (group needs).

##### 1.1.2.1 Affective commitment

Commitment is a driving force that connects a person to a specific course of action that is significant for one or more objectives (Meyer and Herscovitch, 2001). Commitment can be categorized into three aspects: (i) affective commitment, which refers to the emotional bond, sense of belonging, and participation in the organization; (ii) continuance commitment, which involves recognizing the costs of leaving the organization; and (iii) normative commitment, which refers to the sense of obligation to stay with the organization (Meyer et al., 1993). This study focuses on affective commitment because it reflects an individual’s willingness to take a course of action based on feelings of identification and belongingness. The other two dimensions are likely to be driven by cost analysis and obligations rather than psychological state attachment, which is more connected to intrinsic motivation.

Moreover, recent studies on student commitment in higher education institution found out that affective commitment is influenced by the institution’s commitment in students (Cownie, 2019). This implies that, faculty members have contribution on the student feeling of attachment toward their university. In achieving their academic goal, it was found that commitment mediates the relationship between academic resilience and performance (Nigussie Worku and Urgessa Gita, 2024). The study concluded that the overall commitment was moderate and it is significant to explore the sub variables of commitment separately to determine student academic pursuits, implying the need for further research on commitment in relation to other variables like trust and self-efficacy.

##### 1.1.2.2 Collective trust

Collective trust is a crucial component in effective relational and interpersonal relationships and can be in various forms; faculty trust in the principal, clients (parents/students) and school; and clients trust in the faculty, principal and school (Forsyth et al., 2011). According to Forsyth et al. (2011 p.22), collective trust is defined as a stable group property rooted in the shared perceptions and affect about the trustworthiness of another group or individual that emerges out of social exchanges within the group.” In student level it is their willingness to be vulnerable to their supervisors with confidence that the latter party is benevolent, reliable, competent, honest, and open.” It is based on collective individual perceptions of the trust referents stem from a relationship norm. Building from that definition, in this study, collective trust refers to the willingness of students to rely on their supervisors with confidence that the supervisors will act in their interest. Supervisor-student relationships act as fulcrums for student success. It is based on trust, established through reliable communication and clear expectations (Robertson, 2017). Student trust in teachers proves the quality of teacher-student relationships (Mitchell et al., 2018). Additionally, it generates a safe and supportive atmosphere that enables creative development (Robertson, 2017).

Furthermore, trusting students are more likely to willingly conform to institutional regulations (Romeo, 2018). Collective trust in schools has been examined in terms of school effectiveness (Gray et al., 2016), leadership, social capital, and citizenship (Forsyth et al., 2011). Also linked with efficiency, Van Maele et al. (2014) contended that trust “paves the way to progress toward equity and excellence in education (p.26).” Students’ trust in their supervisors includes the belief that they are capable and supportive enough to help them fulfill their educational needs and achieve their goals. Unlike interpersonal trust that relies on psychological aspects such as cognitive, collective trust focuses on social exchanges among members of the group constructed through both psychologically and shared norms (Forsyth et al., 2011). Confidence in others’ ability to fulfill goals promotes relationship factors, such as commitment (Houri et al., 2019) and cooperation (Bryk and Schneider, 2002).

##### 1.1.2.3 Self-efficacy

Self-efficacy is theorized as a person’s trust in their competence to thrive in a particular situation (Bandura, 1977). It also comprises willpower and persistence to conquer impediments that impede the utilization of instinctive abilities to accomplish goals (Kolbe, 2009). Some studies of self-efficacy scholarship have examined it in many domains, such as career effectiveness, performance (Gray et al., 2016), and school experience (Adams et al., 2013; Forsyth et al., 2011). Most researchers strive to understand how self-efficacy affects behavior (Trujillo and Tanner, 2014). Research has indicated that self-efficacy enhances overall work performance and commitment (Ford, 2014; Meyer and Herscovitch, 2001; Meyer and Maltin, 2001). Specifically, studies maintain that self-efficacy in university students is vigorous and influences both achievement and behavior (Na-Nan et al., 2021). The perception of one’s capabilities is shaped by both positive reinforcement and negative feedback regarding one’s performance or ability to execute tasks (Redmond, 2010; Cownie, 2019; Nigussie Worku and Urgessa Gita, 2024).

Research has underscored the significance of exploring factors influencing individuals’ emotional commitment (Cavanagh et al., 2016). Findings indicate that collective trust has a direct influence on commitment (Cavanagh et al., 2018) or serves as a mediating factor in the effect of self-efficacy (Forsyth et al., 2011; Robertson, 2017) on commitment (Ford, 2014; Meyer and Herscovitch, 2001; Meyer and Maltin, 2010). It is also necessary to ascertain whether self-efficacy mediates the relationship between trust and commitment (Chesnut and Burley, 2015). Forsyth et al. (2011) proposed that there exists a “transactional relationship” between self-efficacy and collective trust. Research has demonstrated that self-efficacy and subsequent task performance are enhanced by receiving comprehensive and detailed performance feedback (Beattie et al., 2016). Thus, investigating the influence of this construct is crucial for comprehending students’ motivation toward goal commitment. Consequently, this study proposes the following hypotheses:

*H1: Collective trust positively influences affective commitment.*

Research shows that trust within organizations serves as a mediator between trust in colleagues and organizational outcomes such as affective commitment. This suggests that when employees trust their organization, they enhance their emotional connection and dedication to it (Tan and Lim, 2009). In the context of Chinese agricultural cooperatives, trust in leadership enhances emotional commitment. This effect is further strengthened by the mediation of member participation, indicating that trust in leadership can foster deeper emotional commitment among members (Hao et al., 2024). The relationship between organizational trust and affective commitment is more pronounced in organizations with fewer bureaucratic structures. This implies that, in environments where employees experience less control and trust, their affective commitment is stronger (Gellatly and Withey, 2012). Indicators of trustworthiness within an organization, such as skilled HR professionals and effective information dissemination, lead to higher levels of employee trust. In turn, this trust enhances affective commitment. Additionally, trust in supervisors directly impacts the affective commitment of subordinates, highlighting the importance of trust at various organizational levels (Klimchak et al., 2020). While cognitive trust, based on reliability and competence, is essential for promoting team viability, affective trust, rooted in emotional connections, also has a positive impact, especially in environments with low virtuality. This underscores the significance of both cognitive and affective aspects of trust in fostering commitment (Lhaden et al., 2024).

*H2: Collective trust positively influences self-efficacy.*

Collectivel trust, which includes trust in both colleagues and the organization, has been shown to significantly enhance self-efficacy. This is particularly evident in educational settings, where trust among teachers and between teachers and principals boosts their collective efficacy, thereby positively influencing self-efficacy (Choong et al., 2023; Fiernaningsih and Herijanto, 2021). Research involving employees at a manufacturing firm found that self-efficacy had a more positive impact on job satisfaction, task performance, and organizational citizenship behaviors when organizational trust was high. This suggests that increased organizational trust amplifies the beneficial effects of self-efficacy (Ozyilmaz et al., 2018). The collective efficacy in a group’s capability to reach objectives, known as collective efficacy, can enhance an individual’s self-efficacy (Oldfield et al., 2018). For instance, interventions that enhance collective efficacy also raise self-efficacy as individuals derive a sense of personal control from their group’s perceived capabilities (Jugert et al., 2016). In group contexts, the perception of collective efficacy can directly influence individual task performance, sometimes even more so than self-efficacy alone. This indicates that collective efficacy can strengthen individual self-efficacy and performance (Kellett et al., 2009). Trust in colleagues and the organization serves as a mediator in the relationship between collective efficacy and organizational citizenship behavior (OCB). This mediation suggests that trust within the organization enhances collective efficacy, which subsequently boosts self-efficacy and positive work behaviors (Choong et al., 2023). The concept of trust felt by leaders is essential. When employees feel that their leaders trust them, their occupational self-efficacy increases, leading to an improvement in their in-role and extra-role performance (Zheng et al., 2019).

*H3: Self-efficacy positively influences affective commitment.*

The relationship between self-efficacy and affective organizational commitment is direct and predictive. Individuals exhibiting elevated self-efficacy levels tend to establish robust emotional connections with their organizations (Karatepe et al., 2007; Orgambídez et al., 2019, 2020; Özdemir et al., 2024). This perception of self-efficacy strengthened emotional commitment by increasing work engagement. When employees perceive their own capabilities, their engagement in tasks increases, thereby enhancing their affective commitment (Orgambídez et al., 2019, 2020). Moreover, self-efficacy plays a role in shaping job satisfaction, which subsequently impacts affective commitment. This indicates that a sense of competence and job satisfaction plays a significant role in fostering a stronger emotional connection to the organization (Karatepe et al., 2007; Uma Sankar et al., 2016; Yousaf and Sanders, 2012). Goal clarity and training can influence the connection between self-efficacy and affective commitment. Well-defined goals and efficient training initiatives boost self-efficacy, consequently strengthening emotional commitment (Li and Tsai, 2019). The concept of psychological empowerment, particularly self-efficacy, is essential in nurturing affective commitment. Individuals who perceive themselves as empowered, competent, and appreciated are more inclined to forge robust emotional ties with their organization (Ochoa Pacheco et al., 2023). Individual resources, including self-efficacy, organizational-based self-esteem, and optimism, play a crucial role in predicting affective commitment. These resources empower employees to feel more capable and emotionally engaged in their work (Bon and Shire, 2019).

*H4*: Self-efficacy mediates the relationship between collective trust and affective commitment.

Trust among colleagues and within an organization is essential for developing affective commitment. Trust in organizations serves as a mediator between trust in coworkers and affective commitment, suggesting that trust at various levels interacts to shape organizational outcomes (Tan and Lim, 2009). A shared sense of trust in both colleagues and the organization can boost collective efficacy, which subsequently has a positive effect on organizational citizenship behaviors (OCBs) and affective commitment (Choong et al., 2023; Choong and Ng, 2023). Self-efficacy acts as a mediator between organizational factors and outcomes. For example, it mediates the link between learning orientation and group efficacy, as well as between affective commitment and group efficacy (Li and Tsai, 2019). Collective efficacy, which is closely linked to self-efficacy, mediates the connection between trust and organizational outcomes, including affective commitment (Choong et al., 2023; Choong and Ng, 2023). Self-efficacy amplifies the beneficial effects of trust on affective commitment by enhancing individuals’ confidence in their abilities and belief in the collective strength of their team or organization. This, in turn, reinforces their emotional attachment and commitment to the organization (Choong et al., 2023; Choong and Ng, 2023; Li and Tsai, 2019). Training and clear goals can further influence the mediation effect of self-efficacy, strengthening its impact on the relationship between affective commitment and group efficacy (Li and Tsai, 2019).

*H5*: Demographic variables (age, gender, grade level, and major) moderate the relationship between collective trust, self-efficacy, and affective commitment.

Research indicates that self-efficacy typically increases during adolescence (Shek and Liang, 2017). Additionally, older students in higher education often report higher levels of self-efficacy and intrinsic motivation, which are associated with better academic performance (Jerez, 2024). This suggests that age may positively influence self-efficacy, potentially affecting its relationship with affective commitment. Gender differences in self-efficacy have been observed, with studies showing that gender identity can predict confidence in various abilities such as creative and entrepreneurial skills (Miller and Alvarez Huerta, 2023). This implies that gender might affect the connection between self-efficacy and affective commitment, as self-efficacy levels differ between males and females. The influence of general self-esteem on the relationship between parental trust and learning engagement is affected by students’ college grades (Fute et al., 2023). This indicates that grade level can alter how self-efficacy interacts with other factors, potentially influencing affective commitment. The choice of academic major impacts self-beliefs and career plans, with certain majors fostering greater confidence in specific skills (Miller and Alvarez Huerta, 2023). This suggests that academic major could affect the relationship between self-efficacy and affective commitment, as different fields of study encourage varying levels of self-efficacy.

### 1.2 Conceptual framework

Given the importance of trust and self-efficacy in goal commitment, we hypothesized that they would be positively associated with students’ affective commitment to active learning. We also explored the moderating role of demographic variables (gender, grade, age, and major) on these relationships. Because of the significant role that instructors play in developing and maintaining professional and social relationships (Cownie, 2020), we expected that students who reported high levels of trust in their instructors would be likely to respond more positively and be more engaged in their affective commitment. Likewise, we anticipated that a sense of self-efficacy would be positively associated with students’ affective commitment toward the institution.

**Figure 1:**
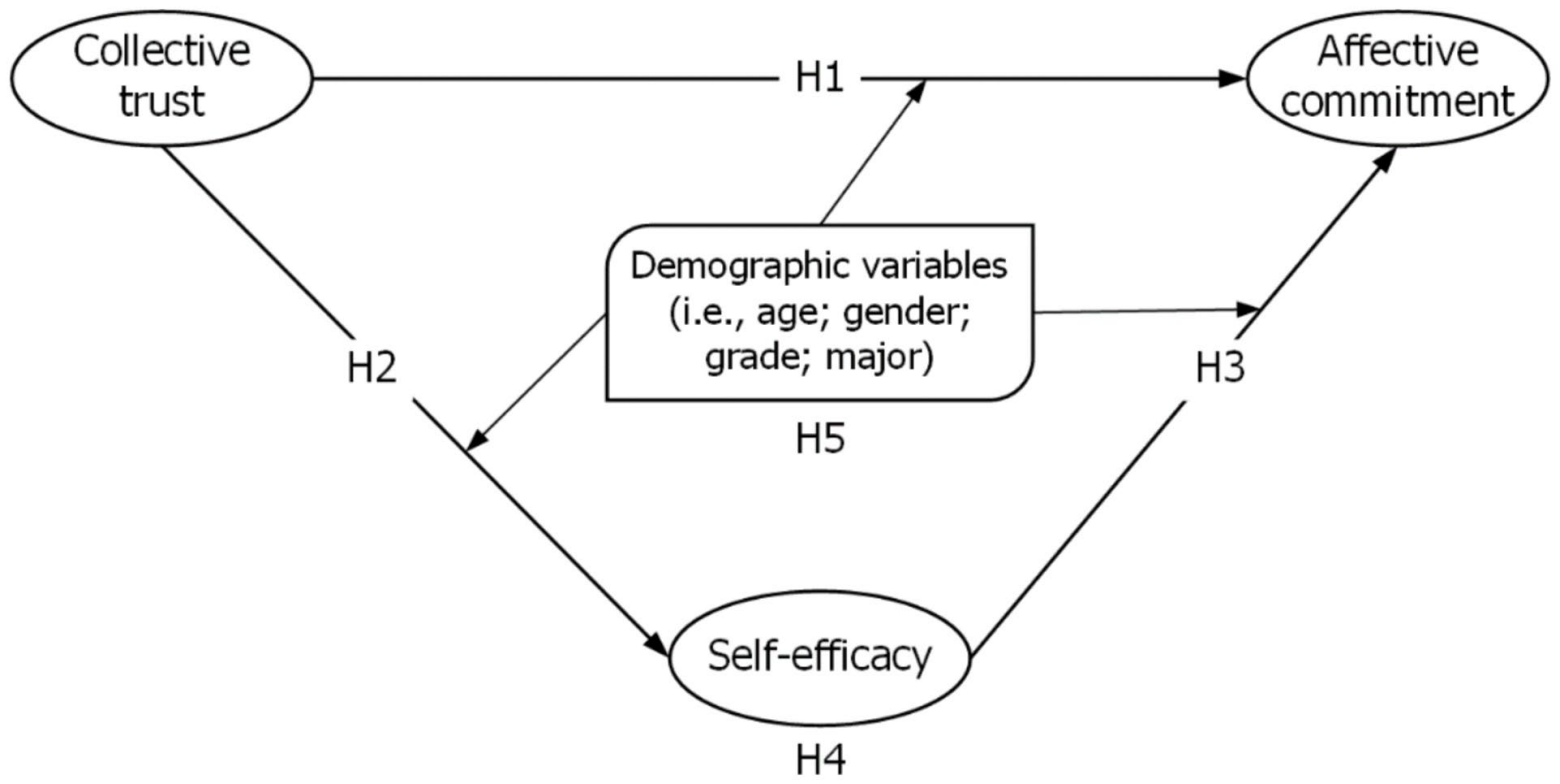
Research model. Authors’ own work.

This study was supported by a conceptual framework signifying affective commitment to the institution as the dependent variable predicted by two independent constructs: collective trust and self-efficacy (see Figure 1). It has been argued that self-efficacy influences the relationship between collective trust and affective commitment toward an institution. Meyer et al. (2004, p. 1002) asserts that “personal values play a role in shaping employee commitment.” Some empirical studies have found collective trust to be a mediating variable in rescuing commitment during a difficult time in the organization (Wang et al., 2018) and have also been theorized to have a transactional relationship with self-efficacy (Forsyth et al., 2011) as well as a determinant of self-efficacy (Robertson, 2017). Trust is directly related to commitment, as indicated in Robertson’s (Robertson, 2017) work. In light of this, we put forth the subsequent hypotheses:

## 2 Methodology

### 2.1 Research design

This study employed a cross-sectional, explanatory, non-experimental design using an online questionnaire administered to a convenience sample of 968 students at Normal University X to test the hypothesized relationships among collective trust, self-efficacy, and affective commitment. Explanatory research is appropriate when the aim is to test theory-driven hypotheses about how and why phenomena operate (Johnson and Christensen, 2017), and it can support causal inference when three conditions are met: (a) a statistical relationship between the predictor and outcome is demonstrated (here assessed through Pearson product–moment partial correlations and structural equation modeling), (b) the putative cause precedes the effect (directionality in this study is justified *a priori* by Relationship Motivation Theory and Social Identity Theory, which posit that trust and social identification shape self-beliefs and emotional attachment), and (c) plausible alternative explanations are controlled (we explicitly included demographic covariates—age, gender, grade level, and major—to account for confounds). Although an experimental design is considered the strongest form for establishing cause-and-effect relationships, it was not adopted because manipulating core independent variables (e.g., supervisory trust or long-standing mentorship practices) was impractical given institutional constraints, ethical considerations, and limited resources and time (Glasofer and Townsend, 2020). Given these practical limits, and because the three criteria for explanatory inference were deliberately addressed through measurement, theory, and covariate control, a cross-sectional explanatory non-experimental design provided the most feasible and methodologically defensible approach for investigating the proposed relationships (Johnson and Christensen, 2017).

### 2.2 Sample

A total sample of 968 (223 male and 745 female) students at Normal University X in China was conveniently drawn from different faculties. Their ages ranged from 18 to 42 years, with a mean of 2.03 and SD = 0.706. Based on the nature of enrolment, students who join the university for the first time take the university entrance examination, “*gaokao*,” and their scores determine which universities they go to. This means that, to obtain admission to a reputable university in China, students must be determined and hard-working. The selected university recruits approximately 3,800 undergraduate and 4,900 graduate students annually from across the country. The diversity of the sample can help to explain the general concepts of self-efficacy, trust, and commitment among Chinese university students. To ensure representation, students from natural science and social science disciplines from undergraduate to graduate levels were invited to fill the questionnaire.

In terms of the demographic profile, based on gender, 745 respondents (77%) were female, significantly outnumbering 223 males (23%). Since it is a ‘Normal’ university that is specialized in grooming teachers, the number of female students exceeds that of male students due to major preferences as argued in a recent study (Xu et al., 2023). Regarding academic majors, 751 respondents (77.6%) were enrolled in the social sciences, while 217 (22.4%) belonged to science-related disciplines. In terms of educational attainment, the majority of respondents were undergraduate students, comprising 487 students (50.3%), followed by 398 master’s students (41.1%) and a smaller proportion of PhD candidates, totaling 83 (8.6%). The age distribution shows that 611 respondents (63.1%) fell within the 19–24 age range, making it the largest group. This was followed by 185 students (19.1%) aged 18 years, 130 (13.5%) in the 25–30 range, and 42 (4.3%) aged 31 years and above. Even though they are from the same country, the diversity of Chinese culture may be represented, as posited by Poort et al. (2022), that “a single-nationality group does not mean all participants have the same cultural background (p.5).”

### 2.3 Data collection procedures and ethical considerations

An online questionnaire, with three scales (comprising 28 measuring items) and demographic information (comprising five variables), was distributed to capture the sense of self-efficacy, collective trust, and affective commitment. The students were approached by Chinese teachers and a research assistant (a university graduate) either through an online messaging application (WeChat) or directly by the researchers. The survey clearly stated the study objectives, assuring that participation was anonymous and voluntary and would not affect their course grades. The participants signed an electronic informed consent form to participate in this study, and no identifiable information was collected. This study followed the ethical guidelines of the Declaration of Helsinki, ensuring that participants’ dignity, rights, safety, and welfare were protected.

### 2.4 Instrumentation

*Commitment:* A subscale of the commitment survey (Allen and Meyer, 1990), modified by Merritt (2012) and containing eight items, was used to measure students’ affective commitment. The wording of these items was modified by replacing “this organization” with “this school.”

*Collective trust:* A collective trust survey adapted from Forsyth et al. (2011) was used to measure students’ trust in their supervisors. This scale originally measures five facets of trustworthiness (benevolence, reliability, honesty, openness, and competence) at the individual level comprising 13 items. It was modified by replacing ‘teachers’ with ‘supervisors.’ Each participant reported his or her view regarding students’ interactions and social exchanges with their supervisors (collective norm) not individual trust in a supervisor. Through interaction with peers, each student has knowledge of the interaction norm with their supervisors, given that they work in groups as supervisees. Other studies that measured collective trust in this manner include Adams (2013), Casper (2012), Ensley (2014).

*Self-efficacy:* Seven items measuring sense of self-efficacy (Chen et al., 2001) were adopted for this study. All survey items were measured using a five-point scale anchored by 1, “strongly disagree,” and 5, “strongly agree,” and the mid-point, 3, labeled as “neutral.” As the study was conducted in a Chinese-speaking environment, all the measures were translated into Chinese and piloted before official use. It is clear from the pilot data that the survey items had greater potency, variance, and relevance.

### 2.5 Data analysis

All questionnaire responses were screened and cleaned prior to hypothesis testing. Missing values were identified and addressed, and two negatively worded items were reverse scored: “Supervisors at this school do not care about students [B3]” and, “I think I could easily become as attached to another school as I am to this one [comt8].” Demographic variables were tabulated, and univariate normality was assessed with skewness and kurtosis. The full sample of 968 cases met the normality criteria and was used in subsequent analyses. We followed the two-step approach to covariance-based structural equation modeling in AMOS 24 (Anderson and Gerbing, 1988). In the measurement phase, we used confirmatory factor analysis to evaluate indicator loadings, internal consistency (via composite reliability and Cronbach’s alpha), convergent validity (via average variance extracted), discriminant validity (via heterotrait–monotrait ratio of correlations [HTMT]), and overall measurement model fit. We judged model fit by normed chi square (χ2/df), Tucker Lewis Index (TLI), Comparative Fit Index (CFI), Root Mean Square Error of Approximation (RMSEA), and Standardized Root Mean Square Residual (SRMR) against conventional benchmarks (χ2/df < 3, TLI and CFI > 0.95, RMSEA < 0.06, SRMR ≤ 0.08) (Schreiber, 2008). Composite mean scores for the three latent constructs were computed from the items retained in the measurement model. Descriptive statistics (e.g., mean, standard deviation) and Pearson partial correlations controlling for age, gender, academic major, and educational attainment were calculated in SPSS version 25 to examine bivariate associations and to indicate the degree to which participants reported collective trust, self-efficacy, and affective commitment. In the structural phase, we tested the hypothesized direct effects. Indirect effects were examined with bootstrap mediation using 2,000 resamples, and moderation by demographic covariates was tested with Hayes’s PROCESS macro for SPSS version 4.2 (Hayes, 2013).

To probe alternative temporal orderings given the cross-sectional design, we estimated several plausible model permutations (see Table 1) and compared the explained variance and path coefficients across specifications. We estimated six permutations (e.g., CT → AC → SE, SE → CT → AC, and AC → SE → CT) and compared the variance explained in the focal outcome for each specification. The hypothesized model (CT → SE → AC) accounted for 50% of the variance in affective commitment (R^2^ = 0.50) and yielded standardized paths of CT → AC = 0.52, CT → SE = 0.42, and SE → AC = 0.31. A reciprocal specification with SE as an antecedent to CT (SE → CT → AC) produced the same *R*^2^ for affective commitment (*R*^2^ = 0.50) and similar coefficients, while several other permutations produced lower explanatory power (*R*^2^ = 0.29–0.43). These checks indicate that alternative orderings are empirically plausible; however, the hypothesized ordering was selected because it is theoretically motivated by Relationship Motivation and Social Identity Theories and because it delivers equivalent or superior explanatory power compared with most alternative permutations. Therefore, we present a unidirectional model while acknowledging that reciprocal dynamics may operate in practice. We recommend future cross-lagged or experimental studies to directly test bidirectional effects.

**Table 1:** Comparative model results.

Model (order)	Focal outcome (DV)	R^2^	Path 1 (A → B)	Path 2 (B → C)	Path 3 (A → C)
CT → SE → AC (hypothesized)	Affective commitment (AC)	0.50	CT → SE = 0.42	SE → AC = 0.31	CT → AC = 0.52
SE → CT → AC	Affective commitment (AC)	0.50	SE → CT = 0.42	CT → AC = 0.52	SE → AC = 0.31
SE → AC → CT	Collective trust (CT)	0.43	SE → AC = 0.53	AC → CT = 0.59	SE → CT = 0.11
AC → SE → CT	Collective trust (CT)	0.43	AC → SE = 0.53	SE → CT = 0.11	AC → CT = 0.59
AC → CT → SE	Self-efficacy (SE)	0.29	AC → CT = 0.65 (R^2^ for CT = 0.42)	CT → SE = 0.14	AC → SE = 0.44
CT → AC → SE	Self-efficacy (SE)	0.29	CT → AC = 0.65	AC → SE = 0.44	CT → SE = 0.14

R^2^ refers to the variance explained in the model’s focal outcome (the final variable in the model order). CT = collective trust; SE = self-efficacy; AC = affective commitment. Values reported are based on the same cross-sectional sample and the same estimation method for comparability. These checks were conducted to probe alternative orderings; they do not establish temporal causality.

## 3 Results

### 3.1 Psychometric properties of the scales

**Figure 2:**
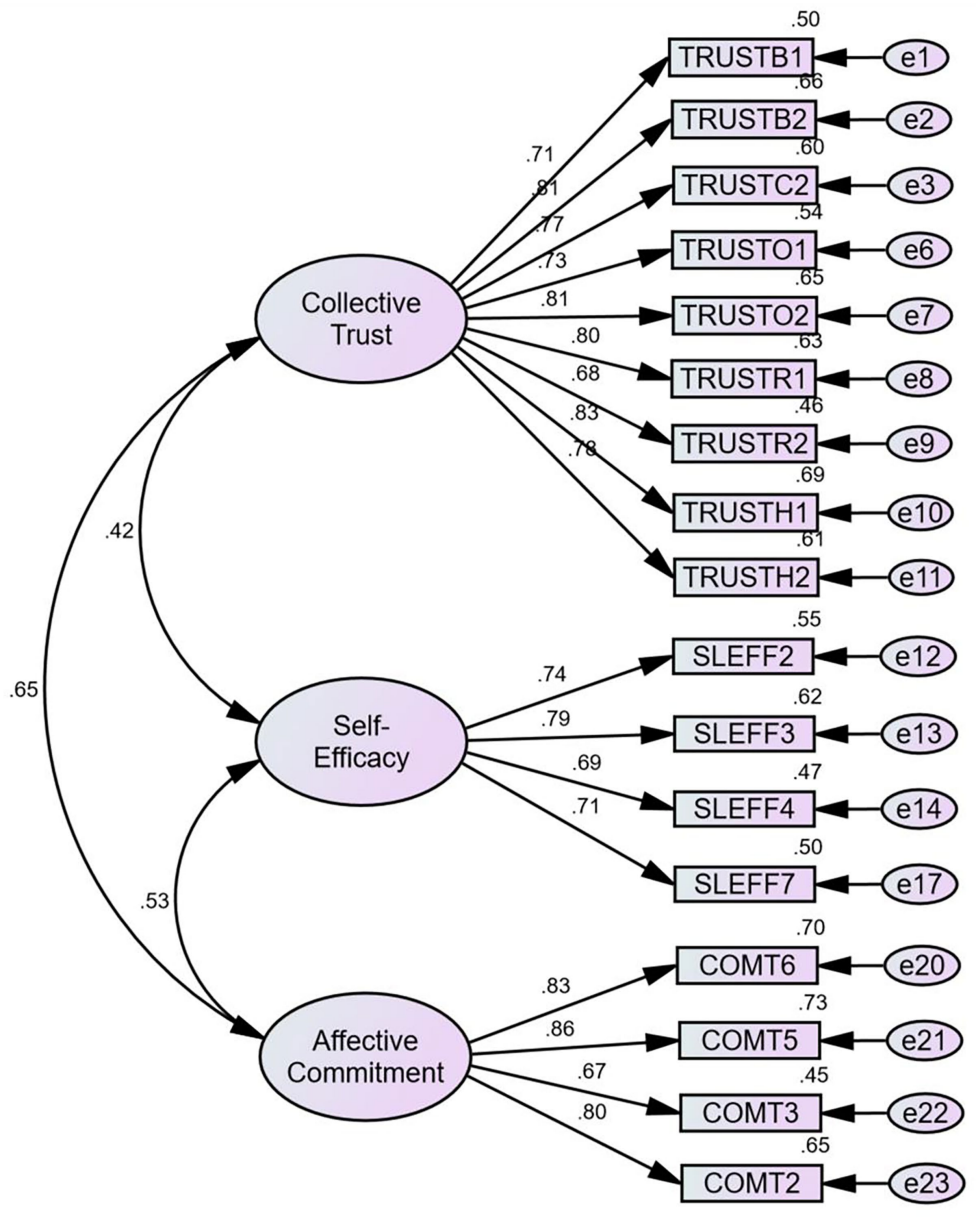
Measurement model comprising three latent constructs. Source: authors’ own work.

Univariate normality and confirmatory factor analysis supported the use of the measures in subsequent modelling. Skewness ranged from −1.58 to −0.10 and kurtosis ranged from 0.02 to 4.72, which fall well within commonly accepted thresholds for normality, where skewness values between −2 and +2 and kurtosis values between −7 and +7 are considered acceptable (Hair et al., 2010). The initial measurement model included 28 items and was estimated using a sample of 968 participants. Eleven items were removed during measurement refinement. Two items were deleted because of very low standardized loadings (B3, loading = −0.102; Comt4, loading = 0.104), and nine additional items were removed because they produced large modification indices indicative of local misfit or cross-loading problems (Comt1 M.I. = 152.165; TrustC3 M.I. = 95.857; SEFF5 M.I. = 67.252; TrustC4 M.I. = 64.971; SEFF6 M.I. = 38.425; Comt8 M.I. = 36.988; Comt7 M.I. = 27.767; SEFF1 M.I. = 22.618; TrustC1 M.I. = 15.11). The resulting 17-item model (see Figure 2) displayed a good fit to the data, as indicated by χ^2^(116) = 339.467, χ^2^/df = 2.926, CFI = 0.976, TLI = 0.972, RMSEA = 0.045, and SRMR = 0.027. All retained indicators had standardized loadings above 0.50 (see Table 2), meeting the conventional acceptability thresholds (Hair et al., 2019).

**Table 2:** Measurement model results.

Constructs and measurement	*ꞵ*	t (significance)	CR	AVE	*α*
Collective trust			0.93	0.59	0.93
B1- Supervisors are always ready to help.	0.71	Fixed			
B2- Students are well cared for at this school.	0.81	24.23***			
C2- Supervisors at this school do a terrific job.	0.77	23.11***			
O1- Supervisors at this school are easy to talk to.	0.73	21.97***			
O2- Supervisors at this school really listen to students.	0.81	24.13***			
R1- Supervisors at this school always do what they are supposed to.	0.80	23.80***			
R2- Students at this school can depend on supervisors for help.	0.68	20.34***			
H1- Supervisors at this school are always honest with me.	0.83	24.71***			
H2- Students can believe what teachers tell them.	0.78	23.40***			
Self-efficacy (SE)			0.82	0.53	0.82
SE2- When facing difficult tasks, I am certain that I will accomplish them.	0.74	Fixed			
SE3- In general, I think that I can obtain outcomes that are important to me	0.79	21.57***			
SE4- I believe I can succeed at almost any endeavor to which I set my mind.	0.69	19.31***			
SE7–7 Even when things are tough, I can perform quite well.	0.71	19.80***			
Affective commitment (Co)			0.87	0.63	0.86
Co2- I feel “emotionally attached” to this school.	0.80	Fixed			
Co3- This school has a great deal of personal meaning for me.	0.67	21.80***			
Co5- I am very happy being a member of this school.	0.86	29.15***			
Co6- I enjoy discussing my school with people outside it.	0.83	28.29***			

*N* = 968. ***p ≤ 0.001; B–benevolence, C–competence, R–reliability, H–honesty, O–openness. ꞵ–standardized regression weight, t–critical ratio, CR–composite reliability, α–Cronbach’s alpha, AVE–average variance extracted. Source: authors’ own work.

The scales’ reliability and construct validity are satisfactory. Composite reliabilities and Cronbach’s alpha coefficients ranged from 0.82 to 0.93 (Table 2), exceeding the recommended minimum of 0.70 (Ali et al., 2018; Fraenkal and Wallen, 2000). Average variance extracted (AVE) values exceeded 0.50 for all constructs (see Table 2), supporting convergent validity (Ali et al., 2018; Fornell and Larcker, 1981). Discriminant validity assessed with the heterotrait–monotrait ratio of correlations produced values between 0.420 and 0.660 well below the 0.85 threshold recommended by Henseler et al. (2015). Partial correlations controlling for age, gender, program of study, major, and year of enrolment indicate that collective trust was moderately associated with affective commitment (*r* = 0.590, *p* < 0.001), collective trust was positively associated with self-efficacy (*r* = 0.393, *p* < 0.001), and self-efficacy was positively associated with affective commitment (*r* = 0.452, *p* < 0.001). These patterns of partial correlations, which control for key demographic covariates, align with theoretical expectations and provide additional evidence of convergent validity before testing the structural model.

### 3.2 Structural equation modeling analysis and hypothesis testing

**Figure 3:**
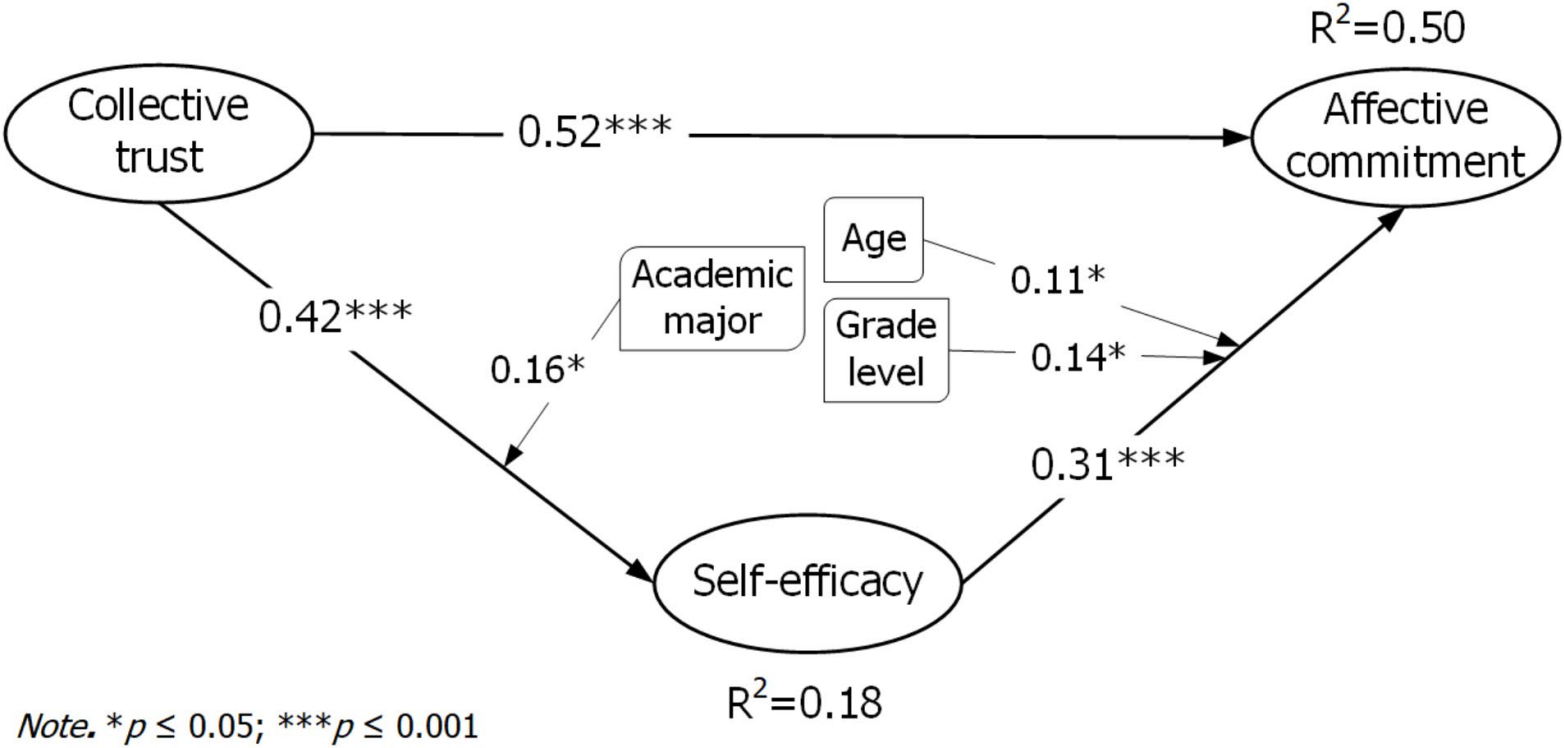
Structural moderated mediation model. Source: authors’ own work.

Structural equation modeling was used to test the hypothesized relationships between collective trust, self-efficacy, and affective commitment (see Figure 3) in AMOS 24. Descriptive composite scores indicated that participants reported high collective trust (*M* = 4.21, SD = 0.57), high affective commitment (*M* = 4.01, SD = 0.69), and moderate self-efficacy (*M* = 3.67, SD = 0.63). Hypothesis 1 proposed that collective trust positively influences affective commitment. The results supported H1, with collective trust positively predicting affective commitment, *β* = 0.517, *t* = 13.922, *p* < 0.001, and an effect size (*f*^2^ = 0.3227) consistent with a moderate effect (see Table 3; Figure 3). Hypothesis 2 proposed that collective trust positively influences self-efficacy. The results supported H2, with collective trust positively predicting self-efficacy, *β* = 0.423, *t* = 10.902, *p* < 0.001, and *f*^2^ = 0.218, indicating a moderate effect (see Table 3; Figure 3). Hypothesis 3 proposed that self-efficacy positively influences affective commitment. The results supported H3 with self-efficacy positively predicting affective commitment, *β* = 0.309, *t* = 8.847, *p* < 0.001, although *f*^2^ = 0.138 falls in the weak effect range (see Table 3; Figure 3). Together, collective trust and self-efficacy explained 49.8 percent of the variance in affective commitment, *R*^2^ = 0.498, and the combined effect size for the predictors on commitment was *f*^2^ = 0.992, indicating a strong overall effect; detailed effect size computations are reported in Table 4.

**Table 3:** Direct path analysis.

Hypotheses	Path description	*ꞵ*	*t*-value	Results
H_1_	Collective trust ➔ affective commitment	0.517	13.922***	Supported
H_2_	Collective trust ➔ self-efficacy	0.423	10.902***	Supported
H_3_	Self-efficacy ➔ affective commitment	0.309	8.847***	Supported

***p ≤ 0.001; source: authors’ own work.

**Table 4:** Computing effect size on self-efficacy and affective commitment.

Effect size of collective trust and self-efficacy on affective commitment					
Independent Variable (IV)	R_I_^2^	R_E_^2^	(R_I_^2^ – R_E_^2^)	$\frac{\left(R_{I}^{2}-R_{E}^{2}\right)}{\left(1-R_{I}^{2}\right)}$	Effect size
Collective trust	0.498	0.336	0.162	0.3227	Moderate
Self-efficacy		0.429	0.069	0.1375	Weak
Collective trust + self-efficacy	0.498	1–0.498 = 0.502		0.498/0.502 = 0.9920	Strong

Effect size of collective trust on self-efficacy				
IV	R_I_^2^	1 – R_I_^2^	$\frac{\left(R_{I}^{2}\right)}{\left(1-R_{I}^{2}\right)}$	Effect size
Collective trust	0.179	0.821	0.218	Moderate

R_I_^2^ (squared correlation for all the independent variables); R_E_^2^ (squared correlation for all independent variables except one); Cohen (f^2^) Less than 0.15 = Weak; 0.15 to 0.35 = Moderate; Greater than 0.35 = Strong. Source(s): Authors’ own creation.

### 3.3 Mediation analysis

Mediation analysis was conducted using a bootstrap procedure with 2,000 resamples, following contemporary recommendations for testing indirect effects (Hayes, 2013). Hypothesis 4 proposed that self-efficacy mediates the relationship between collective trust and affective commitment. The results reported in Table 5 show a significant indirect effect of collective trust on affective commitment through self-efficacy (indirect *β* = 0.098, *p* < 0.001), while the direct effect remained significant when the mediator was included (direct with mediator *β* = 0.490, *p* < 0.001), indicating partial mediation and confirming H4 (see Table 5). The total effect (direct without mediator) was *β* = 0.588 (*p* < 0.001), and the indirect effect therefore accounted for roughly 16.7% of the total effect (0.098/0.588 ≈ 0.167), suggesting that while collective trust has an important direct association with affective commitment, a meaningful portion of its influence operates via enhanced student self-efficacy.

**Table 5:** Results of mediation analysis.

Relationship	Hypothesis	Direct without mediator (*p*-value)	Direct with mediator (*p*-value)	Indirect effect (*p*-value)	Mediation type
Collective trust ➔ self-efficacy ➔ Affective commitment	H_4_	0.588 ^(***)^	0.49 ^(***)^	0.098^***^	Partial mediation

***p ≤ 0.001; source: authors’ own work.

### 3.4 Moderation analysis

Hypothesis 5 proposed that demographic variables (age, gender, grade level, and major) would moderate the relationships among collective trust, self-efficacy, and affective commitment. Moderation tests using Hayes’ PROCESS procedure indicated three significant interaction effects: age significantly moderated the effect of self-efficacy on affective commitment (*β* = 0.114, *p* = 0.008) and grade level significantly moderated the effect of self-efficacy on affective commitment (*β* = −0.141, *p* = 0.002). Academic major significantly moderated the effect of collective trust on self-efficacy (*β* = 0.162, *p* = 0.034). All other tested interactions were non-significant: collective trust to affective commitment for age group (*β* = −0.060, *p* = 0.155), gender (*β* = 0.024, *p* = 0.713), grade level (*β* = −0.002, *p* = 0.966), and major (*β* = 0.982, *p* = 0.177); self-efficacy to affective commitment for gender (*β* = −0.053, *p* = 0.457) and academic major (*β* = −0.041, *p* = 0.603); and collective trust to self-efficacy for age group (*β* = 0.007, *p* = 0.876), gender (*β* = 0.051, *p* = 0.457), and grade level (*β* = −0.064, *p* = 0.148) (see Table 6). Therefore, these results partially support H5.

**Table 6:** Results of moderation analysis.

Moderator	Relationship	Interaction effect *(β)*	*p*-value	Moderation
Age	Collective trust ➔ commitment	−0.060	0.155	No
Gender	Collective trust ➔ commitment	0.024	0.713	No
Grade Level	Collective trust ➔ commitment	−0.002	0.966	No
Major	Collective trust ➔ commitment	0.982	0.177	No
Age	Self-efficacy ➔ commitment	0.114	0.008*	Yes
Gender	Self-efficacy ➔ commitment	−0.053	0.457	No
Grade Level	Self-efficacy ➔ commitment	−0.141	0.002*	Yes
Major	Self-efficacy ➔ commitment	−0.041	0.603	No
Age	Collective trust ➔ self-efficacy	0.007	0.876	No
Gender	Collective trust ➔ self-efficacy	0.051	0.457	No
Grade Level	Collective trust ➔ self-efficacy	−0.064	0.148	No
Major	Collective trust ➔ self-efficacy	0.162	0.034*	Yes

*p ≤ 0.05; source: authors’ own work.

Bold values are significant at *p* ≤ 0.05 or *p* ≤ 0.01.

## 4 Discussions

This study investigated whether collective trust in supervisors and students’ self-efficacy function as mechanisms that help cultivate affective commitment to the university, focusing on a normal Chinese university where supervision commonly combines guided autonomy with group seminars and mentorship practices that foster knowledge sharing and a sense of belonging (Hu and Zhou, 2024; Wang and Byram, 2019). The Chinese supervisory context matters because its blended model of individual mentorship and collective forums creates distinctive relational and social identity processes that make trust and self-beliefs especially salient for student engagement and institutional attachment (Deci and Ryan, 2014; Tajfel and Turner, 2004). We based our hypothesized directional ordering on Relationship Motivation and Social Identity Theories, which articulate the mechanisms by which group-level trust supports competence beliefs and social identification, thereby promoting commitment. Nonetheless, we acknowledge that reciprocal or alternative causal paths are plausible (e.g., individuals with strong self-efficacy may be more likely to perceive supervisors or institutions as trustworthy), and that real-world supervisory relationships may feature mutual reinforcement between trust and efficacy. This study has a few limitations. The cross-sectional design restricts causal inference, the sample was drawn by convenience from a single university and therefore limits generalizability, and the demographic distribution of participants was not proportionate across age, gender, major, and level of study, which may affect the detection and interpretation of moderation effects. Despite these limitations, the study makes a significant contribution by applying integrative theory to an under-examined higher education context, by using rigorous psychometric and structural equation modeling procedures with a large sample to test complex mechanisms, and by establishing an empirical foundation that future longitudinal or multi-site research can build on to inform interventions aimed at strengthening student commitment.

Hypothesis 1, which proposes that collective trust positively influences affective commitment, is supported. Collective trust exerts a moderate-sized effect on affective commitment, and this finding is best understood through group- and identity-based mechanisms, rather than purely individual-level processes. From a relationship-motivation perspective, trust operating at the supervisory or unit level satisfies students’ need for relatedness and psychological safety, thereby facilitating emotional attachment to the institution (Deci and Ryan, 2014). Social identity theory further clarifies that when students perceive their supervisory group or cohort as trustworthy, they are more likely to internalize group norms and incorporate the group into their self-definition, which strengthens their affective commitment to a larger institution (Tajfel and Turner, 2004). These group-level dynamics align with cluster-based evidence showing that trust in leadership or within tight-knit work groups enhances collective commitment in cooperative and team settings (Hao et al., 2024; Tan and Lim, 2009), and that the magnitude of this effect varies systematically across organizational clusters; for example, it is stronger in less bureaucratic, more relational contexts (Gellatly and Withey, 2012). The present results also resonate with findings that both cognitive and affective dimensions of trust matter for commitment, particularly in face-to-face or low-virtuality clusters, where emotional bonds are more readily formed (Dimas et al., 2024; Klimchak et al., 2020). Where prior studies have reported weaker or inconsistent links, those discrepancies can often be traced to differences in the cluster-level context (e.g., virtual teams, high bureaucracy, or weak group identification), which mitigate the relational processes emphasized by relationship motivation and social identity accounts. Taken together, the pattern observed here underscores that interventions aimed at strengthening group-level trust (for example, within supervisory cohorts or seminar groups) are likely to yield meaningful gains in students’ affective commitment, because they operate on social and identity pathways that bind individuals to their institution.

Hypothesis two was confirmed, as collective trust was found to exert a moderate effect on student self-efficacy. This is best interpreted as a group-level relational process rather than a simple individual attribute change. From a Relationship Motivation Theory perspective, trusting supervisory relationships provide autonomy support, corrective feedback, and social persuasion that satisfy students’ needs for competence and relatedness, thereby fostering efficacy beliefs (Bandura, 1977; Deci and Ryan, 2014). Social Identity Theory adds that when students identify with a trustworthy supervisory cohort or seminar group, they gain vicarious learning opportunities and positive social comparisons that raise perceived capability through shared norms and collective efficacy beliefs (Oldfield et al., 2018; Tajfel and Turner, 2004). This cluster-level account aligns with studies showing that trust within educational and organizational teams bolsters collective efficacy and, in turn, individual self-efficacy and performance (Choong and Ng, 2023; Jugert et al., 2016; Kellett et al., 2009), and with evidence that leader-level trust amplifies the beneficial effects of personal efficacy on outcomes (Ozyilmaz et al., 2018; Zheng et al., 2019). Where prior work has reported weaker links, differences in cluster contexts, such as high bureaucracy, low face-to-face contact, or limited opportunities for mastery experiences, help explain divergence because they reduce the relational and vicarious pathways through which trust operates (Gellatly and Withey, 2012). In short, the present results suggest that enhancing trust at the supervisory or cohort level is a plausible lever for strengthening student self-efficacy, especially in settings that preserve rich interpersonal interactions and opportunities for shared success.

Although Hypothesis 3 was supported, the effect of self-efficacy on affective commitment was statistically significant but weak, which invites an in-depth interpretation rooted in relationship and identity based mechanisms. From a Relationship Motivation Theory perspective, self-efficacy fosters engagement and goal-directed behavior that can translate into stronger emotional bonds with the institution via increased task involvement and satisfaction (Deci and Ryan, 2014; Karatepe et al., 2007). Social Identity Theory complements this view by suggesting that efficacy beliefs formed within a trusted supervisory or cohort context may be internalized as part of students’ social identity, thereby bolstering commitment through vicarious learning and shared norms (Oldfield et al., 2018; Tajfel and Turner, 2004). The relatively small direct effect observed here is consistent with cluster-level evidence indicating that individual resources, such as self-efficacy, often exert larger effects on performance and engagement than on affective attachment when group-level factors (for example, collective trust or collective efficacy) are salient (Choong et al., 2023; Kellett et al., 2009). In other words, self-efficacy may operate more potently as a proximal driver of behavior and satisfaction, which then feeds into commitment indirectly (Orgambídez et al., 2019, 2020; Uma Sankar et al., 2016), or its impact may be conditional on supportive conditions, such as goal clarity, training, and empowering leadership (Li and Tsai, 2019; Ochoa Pacheco et al., 2023). Where prior studies report stronger self-efficacy–commitment links, differences in cluster contexts (e.g., settings with less cohesive supervisory groups or weaker institutional trust) may allow individual beliefs to explain more variance. Conversely, in relationally rich clusters, such as the present sample, group-level trust appears to capture much of the variance in affective attachment, leaving a smaller but still meaningful role for self-efficacy (Bon and Shire, 2019; Gellatly and Withey, 2012). Practically, this suggests that interventions to enhance commitment should not rely on boosting individual confidence alone but should pair self-efficacy development with efforts to strengthen supervisory trust and clear competence-building experiences.

Hypothesis 4 predicted that self-efficacy would mediate the relationship between collective trust and affective commitment, and the pattern of partial mediation observed suggests that self-efficacy transmits a meaningful but not exclusive portion of the influence of trust on students’ attachment to their university. Framed at the cluster level, this result is consistent with Relationship Motivation Theory, which posits that trust-laden supervisory contexts supply autonomy support, feedback, and social persuasion that bolster students’ competence beliefs, and with Social Identity Theory, which suggests that identification with a trustworthy supervisory cohort fosters vicarious learning and internalization of group norms that raise efficacy and, in turn, commitment (Deci and Ryan, 2014; Tajfel and Turner, 2004). We adopted this directional ordering on theoretical grounds; however, reciprocal or alternative causal paths are plausible. Students with higher self-efficacy may be more inclined to perceive supervisors and institutions as trustworthy, and real-world supervisory relationships likely feature mutual reinforcement between trust and self-efficacy. The findings of this study align with cluster-focused work showing that trust promotes collective efficacy and that collective and individual efficacy operate as mechanisms linking organizational trust to positive outcomes (Choong et al., 2023; Choong and Ng, 2023; Li and Tsai, 2019), echoing Forsyth et al. (2011) notion of a transactional relationship between trust and efficacy. At the same time, partial mediation implies that additional pathways beyond self-efficacy, such as perceived organizational support, satisfaction, or belongingness, may carry portions of trust’s effect on affective commitment, and contextual moderators (e.g., goal clarity, training, or bureaucratic structure) can shape the relative importance of these pathways (Gellatly and Withey, 2012; Li and Tsai, 2019). The results indicate that interventions should combine group-level trust-building with direct efficacy-enhancing practices rather than relying on either approach alone. Future longitudinal or experimental multi-cluster studies should explicitly test bidirectional models and alternative mediators to adjudicate causal ordering and boundary conditions.

Hypothesis 5 received only partial support, and moderation tests identified three significant interactions. Age moderated the effect of self-efficacy on affective commitment, grade level moderated the same path in the opposite direction, and academic major moderated the effect of collective trust on self-efficacy, while the remaining interactions were non-significant. These patterns suggest that the translation of personal efficacy into institutional attachment is contingent on cohort- and stage-related factors: older students may more readily convert efficacy into affective commitment because developmental maturation and clearer vocational identities strengthen the motivational link between competence beliefs and organizational attachment (Jerez, 2024; Shek and Liang, 2017), a point that dovetails with Relationship Motivation Theory’s emphasis on how evolving autonomy and relatedness needs shape motivation (Deci and Ryan, 2014). In contrast, the negative moderation by grade level implies that advanced students (e.g., master’s and doctoral cohorts) may exhibit a weaker efficacy to commitment translation, perhaps because higher-year students develop differentiated role identities, external career attachments, or task-focused orientations that attenuate emotional ties to the institution (Bon and Shire, 2019; Fute et al., 2023). The major-based moderation of collective trust on self-efficacy indicates disciplinary cluster effects: in some program cultures, such as those that emphasize close mentorship, collaborative supervision, or applied group work, trust within supervisory clusters more strongly fosters individual efficacy, consistent with evidence that team- and leader-level trust amplifies collective and individual efficacy (Choong and Ng, 2023; Kellett et al., 2009) and with the literature on discipline-specific socialization regimes (Miller and Alvarez Huerta, 2023). That most demographic interactions were non-significant further indicates that the primary pathways (*collective trust → affective commitment; collective trust → self-efficacy; self-efficacy → affective commitment*) are generally robust across subgroups, even as certain cohort- or discipline-specific boundary conditions modulate effect strength; these complex moderation results therefore point to the pragmatic value of tailoring trust- and efficacy-enhancing interventions by student stage and program cluster, while noting that uneven subgroup sizes may have limited power to detect additional moderating effects.

### 4.1 Theoretical and practical implications

This study advances theory by integrating Relationship Motivation Theory and Social Identity Theory to show how trust operating at the supervisory/cohort level functions as both a direct social glue and an indirect catalyst for student attachment via self-efficacy. By modelling trust as a collective, group-level resource rather than solely an interpersonal attribute, the findings underscore the importance of cluster-level processes (e.g., supervisory cohorts, seminar groups) in shaping motivational states and institutional identification. The partial mediation observed highlights that self-efficacy is an important but not exclusive pathway linking trust to affective commitment, which suggests that theoretical models of student engagement should accommodate multiple parallel mechanisms (for example, perceived organizational support or belongingness) and boundary conditions. Finally, the moderation results point to meaningful heterogeneity across cohorts and disciplinary clusters, implying that theory must account for developmental stages and program-specific socialization as moderators of how relational resources translate into psychological outcomes. Therefore, future theoretical work should test dynamic, multilevel formulations, and longitudinal specifications to unpack temporal and contextual contingencies.

Universities can translate these insights into concrete, time-bound actions to strengthen trust and student self-efficacy. First, implement a pilot supervisory-trust program in two departments within 12 months that includes supervisor feedback training, structured group seminars, and transparent communication protocols; success indicator: at least 80% supervisor completion of training and a 10% improvement in departmental student trust scores at the 12-month follow-up. Second, deploy a self-efficacy enhancement series (six evidence-based workshops per semester focused on mastery experiences, goal setting, and social persuasion) and measure impact with a pre/post self-efficacy scale; target a meaningful improvement (for example, a 0.3 standard-deviation increase) within 6 months of program start. Third, require that each supervisor cohort hold regular group mentorship sessions (biweekly or monthly depending on program size) and aim for 75% student attendance and supervisor participation within 9 months. Fourth, establish a monitoring and evaluation system: administer an annual campus survey of collective trust, self-efficacy, and affective commitment; publish results to stakeholders; and use the data to refine interventions. The target is complete coverage of all faculties within 18 months. Finally, targeted, cluster-sensitive actions (e.g., orientation and transition support for early year students and career-integration mentorship for advanced students) should be adopted with the explicit aim of reducing observed subgroup gaps in self-efficacy or commitment by 50% within 18 months. These recommendations are specific, measurable, achievable, relevant to institutional goals, and time-bound, and they can be piloted, evaluated, and scaled so that interventions operate on both group-level trust pathways and individual efficacy mechanisms that jointly foster durable student commitment.

### 4.2 Conclusion

This study examined whether collective trust in supervisors predicts affective commitment, tested self-efficacy as the mediating mechanism linking collective trust to affective commitment, and assessed demographic variables (age, gender, grade level, and major) as moderators of the relationships between collective trust, self-efficacy, and affective commitment. Using a cross-sectional, explanatory, non-experimental design with a convenience sample of 968 students and a two-step covariance-based structural equation modeling approach in AMOS 24, we refined the measurement model, tested direct paths, estimated indirect effects via bootstrap mediation (2,000 resamples), and assessed moderation using Hayes’s PROCESS. The results showed that collective trust positively predicted both affective commitment and self-efficacy, with moderate effect sizes, whereas self-efficacy positively predicted affective commitment with a weak effect size. Self-efficacy also partially mediated the link between collective trust and affective commitment, and demographic moderators produced only partial support. Together, collective trust and self-efficacy accounted for approximately 50 per cent of the variance in affective commitment, indicating a strong combined influence. Key limitations include the cross-sectional design that limits causal inference, convenience sampling from a single university that constrains generalizability, and uneven subgroup sizes that may have reduced the power to detect moderation. These caveats require caution when interpreting directionality and subgroup effects. Future research should prioritize longitudinal or experimental designs, cross-validation of the measurement model across multiple institutions and cultural contexts, and examination of additional mediators and boundary conditions (e.g., perceived organizational support, goal clarity, and collective efficacy) to better map the mechanisms through which trust and efficacy translate into durable institutional attachment.

## Data Availability

The raw data supporting the conclusions of this article will be made available by the authors, without undue reservation.

## References

[ref1] Adams C. M. (2013). Collective trust: a social indicator of instructional capacity. J. Educ. Adm. 51, 363–382. doi: 10.1108/09578231311311519

[ref3] Ali F. Rasoolimanesh S. M. Sarstedt M. Ringle C. M. Ryu K. (2018). An assessment of the use of partial least squares structural equation modeling (PLS-SEM) in hospitality research. Int. J. Contemp. Hospit. Manag. 30, 514–538. doi: 10.1108/IJCHM-10-2016-0568

[ref4] Ali P. A. Watson R. (2016). Postgraduate research students’ and their supervisors’ attitudes towards supervision. Int. J. Doctoral Stud. 11, 227–241. doi: 10.28945/3541

[ref5] Allen N. J. Meyer J. P. (1990). The measurement and antecedents of affective, continuance and normative commitment to the organization. J. Occup. Psychol. 63, 1–18. doi: 10.1111/j.2044-8325.1990.tb00506.x

[ref6] Anderson J. C. Gerbing D. W. (1988). Structural equation modelling in practice: A review and recommended two-step approach. Psychol. Bull. 103, 411–423. doi: 10.1037/0033-2909.103.3.411

[ref7] Arriaga X. B. Agnew C. R. (2001). Being committed: affective, cognitive, and conative components of relationship commitment. Personal. Soc. Psychol. Bull. 27, 1190–1203. doi: 10.1177/0146167201279011

[ref8] Bandura A. (1977). Self-efficacy: toward a unifying theory of behavioral change. Psychol. Rev. 84, 191–215. doi: 10.1037/0033-295X.84.2.191, PMID: 847061

[ref9] Beattie S. Woodman T. Fakehy M. Dempsey C. (2016). The role of performance feedback on the self-efficacy–performance relationship. Sport Exerc. Perform. Psychol. 5, 1–13. doi: 10.1037/spy0000051

[ref10] Bon A. T. Shire A. M. (2019). The impacts of second order construct of personal resources on employees’ job performance and the mediating role of affective commitment: SEM analysis approach. Proc. Int. Conf. Ind. Eng. Oper. Manage, 652–664. Available at: https://ieomsociety.org/pilsen2019/papers/193.pdf

[ref11] Bowden J. Wood L. (2011). Sex doesn’t matter: the role of gender in the formation of student-university relationships. J. Mark. High. Educ. 21, 133–156. doi: 10.1080/08841241.2011.623731

[ref12] Brower H. H. Lester S. W. Korsgaard M. A. Dineen B. R. (2009). A closer look at trust between managers and subordinates: understanding the effects of both trusting and being trusted on subordinate outcomes. J. Manage. 35, 327–347. doi: 10.1177/0149206307312511

[ref13] Bryk A. S. Schneider B. (2002). Trust in Schools: A Core resource for improvement. New York: Russell Sage Foundation.

[ref14] Casper D. C. (2012) The relationship between collective student trust and student achievement [PhD dissertation, university of Oklahoma]. Available online at: https://shareok.org/server/api/core/bitstreams/a1deeab9-0bb3-4600-a9ad-dbd08e966f42/content#:~:text=Collective%20student%20trust%20was%20the,poverty%20schools%20with%20low%20trust.

[ref15] Cavanagh A. J. Aragón O. R. Chen X. Couch B. A. Durham M. F. Bobrownicki A. . (2016). Student buy-in to active learning in a college science course. CBE Life Sci. Educ. 15, 1–9. doi: 10.1187/cbe.16-07-0212 PMC5132373 27909026

[ref16] Cavanagh A. J. Chen X. Bathgate M. Frederick J. Hanauer D. I. Graham M. J. (2018). Trust, growth mindset, and student commitment to active learning in a college science course. CBE Life Sci. Educ. 17:ar10. doi: 10.1187/cbe.17-06-0107, PMID: 29378750 PMC6007784

[ref17] Chen G. Gully S. M. Eden D. (2001). Validation of a new general self-efficacy scale. Organ. Res. Methods 4, 62–83. doi: 10.1177/109442810141004

[ref18] Chesnut S. R. Burley H. (2015). Self-efficacy as a predictor of commitment to the teaching profession: a meta-analysis. Educ. Res. Rev. 15, 1–16. doi: 10.1016/j.edurev.2015.02.001

[ref19] Choong Y.-O. Lau T.-C. Ng L.-P. (2023). Collective efficacy among schoolteachers: influences on the relationship between trust and organisational citizenship behaviour. J. Psychol. Afr. 33, 594–603. doi: 10.1080/14330237.2023.2279373

[ref20] Choong Y.-O. Ng L.-P. (2023). The effects of trust on efficacy among teachers: the role of organizational citizenship behaviour as a mediator. Curr. Psychol. 42, 19087–19100. doi: 10.1007/s12144-022-03067-1

[ref21] Cownie F. (2019). ‘What drives students’ affective commitment towards their university?’. J. Furth. High. Educ. 43, 674–691. doi: 10.1080/0309877X.2017.1394988

[ref22] Cownie F. (2020). How commitment influences students’ conversations about higher education. J. Furth. High. Educ. 44, 1401–1418. doi: 10.1080/0309877X.2019.1690641

[ref23] Cownie F. Bradney A. (2017). An examined life: research into university legal education in the United Kingdom and the journal of law and society. J Law Soc 44, S129–S143. doi: 10.1111/jols.12053

[ref25] Deci E. L. Ryan R. M. (2014). “Autonomy and need satisfaction in close relationships: relationships motivation theory” in Human motivation and interpersonal relationships. ed. Weinstein N. (Springer Netherlands), 53–73.

[ref24] Deci E. L. Ryan R. M. (2012). “Self-determination theory” in Handbook of theories of social psychology. eds. Van Lange P. A. M. Kruglanski A. W. Higgins E. T., vol. 1. 1st ed (Dordrecht, Netherlands: Springer), 416–437. doi: 10.1007/978-94-017-8542-6_3

[ref26] Dimas I. D. Rebelo T. Lourenço P. R. Alves M. P. (2024). When affective commitment leads to viability: the role of trust as a mediator and virtuality as a moderator. Eur. Manage. J. S0263237324001452. doi: 10.1016/j.emj.2024.10.005

[ref27] Ensley J. M. (2014) Academic optimism and collective student trust of teachers: A test of the relationship in urban schools [PhD dissertation, university of Oklahoma]. 1–82. Available online at: https://shareok.org/items/2b762a6f-b40b-4113-b921-bb3d5002dc21

[ref28] Fiernaningsih N. Herijanto P. (2021). Effect of relational trust and job autonomy on self efficacy and innovative behavior. Acad. Strateg. Manag. J. 20, 1–12.

[ref29] Ford T. G. (2014). “Trust, control, and comprehensive school reform: investigating growth in teacher-teacher relational Trust in Success for all schools” in Trust and school life: The role of Trust for Learning, teaching, leading, and bridging. eds. Van Maele D. Forsyth P. B. Van Houtte M. (Springer, Dordrecht: Springer Netherlands), 229–258.

[ref30] Fornell C. Larcker D. F. (1981). Evaluating structural equation models with unobservable variables and measurement error. J. Mark. Res. 18, 39–50. doi: 10.2307/3151312

[ref31] Forsyth P. Adams C. Hoy W. (2011). Collective trust: Why schools can’t improve without it. New York, USA: Teachers College Press.

[ref32] Fraenkal J. R. Wallen N. E. (2000). How to design and evaluate research in education. Boston, MA: McGraw-Hill.

[ref33] Fute A. Sun B. Oubibi M. (2023). General self-esteem as the mechanism through which early-childhood parental trust and support affect adolescents’ learning behavior: a moderated mediation model. Inquiry 60, 1–9. doi: 10.1177/00469580231152076, PMID: 36786367 PMC9932760

[ref34] Gellatly I. R. Withey M. J. (2012). Organisational trust, affective commitment and bureaucratic control. J. Trust Res. 2, 31–52. doi: 10.1080/21515581.2012.659936

[ref35] Glasofer A. Townsend A. B. (2020). Determining the level of evidence: nonexperimental research designs. Nurs. Crit. Care 15, 24–27. doi: 10.1097/01.CCN.0000612856.94212.9b 33953103

[ref36] Gray G. McGuinness C. Owende P. Hofmann M. (2016). Learning factor models of students at risk of failing in the early stage of tertiary education. J. Learn. Analytics 3, 330–372. doi: 10.18608/jla.2016.32.20

[ref37] Hair J. Black W. C. Babin B. J. Anderson R. E. (2010). Multivariate data analysis. 7th Edn. Upper Saddle River, New Jersey: Pearson Educational International.

[ref38] Hair J. F. Jr. Black W. C. Babin B. J. Anderson R. E. (2019). Multivariate data analysis. In Book (Eighth, 87. England: Cengage Learning EMEA.

[ref39] Hao J. Bijman J. Heijman W. Gao M. (2024). The effect of trust and social pressure on member commitment in agricultural cooperatives – evidence from China. Ann. Public Coop. Econ. 95, 919–944. doi: 10.1111/apce.12467

[ref40] Hayes A. F. (2013). Introduction to mediation, moderation, and conditional process analysis: A regression-based approach. New York, NY: Guilford Press.

[ref41] Hemer S. R. (2012). Informality, power and relationships in postgraduate supervision: supervising PhD candidates over coffee. High. Educ. Res. Dev. 31, 827–839. doi: 10.1080/07294360.2012.674011

[ref42] Henseler J. Ringle C. M. Sarstedt M. (2015). A new criterion for assessing discriminant validity in variance-based structural equation modeling. J. Acad. Mark. Sci. 43, 115–135. doi: 10.1007/s11747-014-0403-8

[ref43] Houri A. K. Thayer A. J. Cook C. R. (2019). Targeting parent trust to enhance engagement in a school-home communication system: a double-blind experiment of a parental wise feedback intervention. School Psychol. 34, 421–432. doi: 10.1037/spq0000318, PMID: 31294599

[ref44] Hu Y. Zhou Z. (2024). Ready to learn from your students? Chinese undergraduate students’ experiences of informal reverse mentoring. Future Educ. Res. 2, 49–64. doi: 10.1002/fer3.17

[ref45] Jerez E. (2024). Exploring the contribution of student engagement factors to mature-aged students’ persistence and academic achievement during the first year of university. J. Contin. High. Educ. 72, 304–319. doi: 10.1080/07377363.2023.2279797

[ref46] Johnson B. Christensen L. B. (2017). Educational research: Quantitative, qualitative, and mixed approaches. Sixth Edn. Thousand Oaks, California: SAGE Publications, Inc.

[ref47] Jugert P. Greenaway K. H. Barth M. Büchner R. Eisentraut S. Fritsche I. (2016). Collective efficacy increases pro-environmental intentions through increasing self-efficacy. J. Environ. Psychol. 48, 12–23. doi: 10.1016/j.jenvp.2016.08.003

[ref48] Karatepe O. M. Arasli H. Khan A. (2007). The impact of self-efficacy on job outcomes of hotel employees: evidence from northern Cyprus. Int. J. Hosp. Tour. Adm. 8, 23–46. doi: 10.1300/J149v08n04_02

[ref49] Kellett J. B. Humphrey R. H. Sleeth R. G. (2009). Career development, collective efficacy, and individual task performance. Career Dev. Int. 14, 534–546. doi: 10.1108/13620430910997286

[ref50] Klimchak M. Ward Bartlett A. K. MacKenzie W. (2020). Building trust and commitment through transparency and HR competence: a signaling perspective. Pers. Rev. 49, 1897–1917. doi: 10.1108/PR-03-2019-0096

[ref51] Kolbe K. (2009). Self-efficacy results from exercising control over personal cognitive strengths. Available at: https://e.kolbe.com/knol/index.html (Accessed October 6, 2025).

[ref52] Lee A. (2018). How can we develop supervisors for the modern doctorate? Stud. High. Educ. 43, 878–890. doi: 10.1080/03075079.2018.1438116

[ref0010] Lhaden K. Dimas I. D. Rebelo T. Lourenço P. R. Alves M. P. (2024). When affective commitment leads to viability: The role of trust as a mediator and virtuality as a moderator. European Management Journal S0263237324001452.

[ref53] Li C.-Y. Tsai C.-Y. (2019). Multilevel study of factors for cultivating self-efficacy in the online game industry. J. Manage. Organ. 25, 672–694. doi: 10.1017/jmo.2017.22

[ref54] Mercurio Z. A. (2015). Affective commitment as a Core essence of organizational commitment: an integrative literature review. Hum. Resour. Dev. Rev. 14, 389–414. doi: 10.1177/1534484315603612

[ref55] Merritt S. M. (2012). The two-factor solution to Allen and Meyer’s (1990) affective commitment scale: effects of negatively worded items. J. Bus. Psychol. 27, 421–436. doi: 10.1007/s10869-011-9252-3

[ref56] Meyer J. P. Allen N. J. Smith C. A. (1993). Commitment to organizations and occupations: extension and test of a three-component conceptualization. J. Appl. Psychol. 78, 538–551. doi: 10.1037/0021-9010.78.4.538

[ref57] Meyer J. P. Becker T. E. Vandenberghe C. (2004). Employee commitment and motivation: A conceptual analysis and integrative model. J. Appl. Psychol. 89, 991–1007. doi: 10.1037/0021-9010.89.6.991, PMID: 15584837

[ref58] Meyer J. P. Herscovitch L. (2001). Commitment in the workplace: toward a general model. Hum. Resour. Manage. Rev. 11, 299–326. doi: 10.1016/S1053-4822(00)00053-X

[ref59] Meyer J. P. Maltin R. R. (2010). Employee commitment and well-being: a critical review, theoretical framework and research agenda. J. Vocat. Behav 77, 323–337.

[ref60] Miller A. L. Alvarez Huerta P. (2023). Exploring the role of gender identity and academic major in skill confidence and entrepreneurial career plans. Entrep. Educ. 6, 295–317. doi: 10.1007/s41959-023-00101-6

[ref61] Mitchell R. M. Kensler L. Tschannen-Moran M. (2018). Student trust in teachers and student perceptions of safety: positive predictors of student identification with school. Int. J. Leadersh. Educ. 21, 135–154. doi: 10.1080/13603124.2016.1157211

[ref62] Na-Nan K. Kanthong S. Joungtrakul J. (2021). An empirical study on the model of self-efficacy and organizational citizenship behavior transmitted through employee engagement, organizational commitment and job satisfaction in the Thai automobile parts manufacturing industry. J. Open Innov. Technol. Mark. Complex. 7:1–19. doi: 10.3390/joitmc7030170

[ref9002] Nigussie Worku B. Urgessa Gita D. (2024) Students’ academic culture: the mediating role of academic commitment in the relationship between academic resilience and academic performance of university students, Cogent Education, 11:2377004. doi: 10.1080/2331186X.2024.2377004

[ref63] O’Doherty K. C. (2023). Trust, trustworthiness, and relationships: ontological reflections on public trust in science. J. Responsib. Innov. 10:2091311. doi: 10.1080/23299460.2022.2091311

[ref64] Ochoa Pacheco P. Coello-Montecel D. Tello M. (2023). Psychological empowerment and job performance: examining serial mediation effects of self-efficacy and affective commitment. Admin. Sci. 13:1–22. doi: 10.3390/admsci13030076

[ref65] Oldfield B. J. Clark B. W. Mix M. C. Shaw K. C. Serwint J. R. Desai S. V. . (2018). Two novel urban health primary care residency tracks that focus on community-level structural vulnerabilities. J. Gen. Intern. Med. 33, 2250–2255. doi: 10.1007/s11606-017-4272-y, PMID: 29299817 PMC6258596

[ref66] Orgambídez A. Borrego Y. Vázquez-Aguado O. (2019). Self-efficacy and organizational commitment among Spanish nurses: the role of work engagement. Int. Nurs. Rev. 66, 381–388. doi: 10.1111/inr.12526, PMID: 31184381

[ref67] Orgambídez A. Borrego Y. Vázquez-Aguado O. (2020). Linking self-efficacy to quality of working life: the role of work engagement. West. J. Nurs. Res. 42, 821–828. doi: 10.1177/0193945919897637, PMID: 31941420

[ref68] Özdemir M. Küçükçene M. Abaslı K. Pektaş V. Ayhan E. (2024). The effect of empowering leadership and teacher autonomy on affective commitment: the mediating role of teacher self-efficacy. Egit. ve Bilim 49, 201–224. doi: 10.15390/EB.2024.12663

[ref69] Ozyilmaz A. Erdogan B. Karaeminogullari A. (2018). Trust in organization as a moderator of the relationship between self-efficacy and workplace outcomes: a social cognitive theory-based examination. J. Occup. Organ. Psychol. 91, 181–204. doi: 10.1111/joop.12189

[ref70] Poort I. Jansen E. Hofman A. (2022). Does the group matter? Effects of trust, cultural diversity, and group formation on engagement in group work in higher education. High. Educ. Res. Dev. 41, 511–526. doi: 10.1080/07294360.2020.1839024

[ref71] Redmond B. F. (2010). Self-efficacy theory: Do I think that I can succeed in my work? Work attitudes & motivations. The Pennsylvania state University, World Campus.

[ref72] Roberts L. D. Seaman K. (2018). Students’ experiences of undergraduate dissertation supervision. Front. Educ. 3:109. doi: 10.3389/feduc.2018.00109

[ref73] Robertson M. J. (2017). Trust: the power that binds in team supervision of doctoral students. High. Educ. Res. Dev. 36, 1463–1475. doi: 10.1080/07294360.2017.1325853

[ref74] Romeo L. (2018). The discipline gap: what’s trust got to do wiith it? Teach. Coll. Rec. 120, 1–30. doi: 10.1177/016146811812001107

[ref75] Schreiber J. B. (2008). Core reporting practices in structural equation modeling. Res. Social Adm. Pharm. 4, 83–97. doi: 10.1016/j.sapharm.2007.04.003, PMID: 18555963

[ref76] Seth M. K. Bhuyan S. (2023). Teacher- student relationship of university students in relation to their academic achievement. Int. Res. J. Modern. Eng. Technol. Sci. 5:3684–3692. doi: 10.56726/IRJMETS42562, PMID: 41003252

[ref77] Shek D. T. L. Liang L.-Y. (2017). “A longitudinal study of self-efficacy in Chinese students in Hong Kong” in Posit. Youth development: Long term effects in a Chinese. Program (Hauppauge: Nova Science Publishers, Inc.; Scopus), 61–82.

[ref9001] Sporn B. Godonoga A. (2024). Higher education institutions as change agents in society: perspectives on adaptation and impact. European Journal of Higher Education, 14, 1–9. doi: 10.1080/21568235.2024.2412764

[ref78] Tahir I. M. Ghani N. A. Atek E. S. E. Manaf Z. A. (2012). Effective supervision from research students’ perspective. Int. J. Educ. 4, 211–222. doi: 10.5296/ije.v4i2.1531

[ref79] Tajfel H. Tunner C. J. (2004). “The social identity theory of intergroup behavior” in Political psychology. ed. Jost J. S. J. T. . 1st ed (New York: Psychology Press), 18. doi: 10.4324/9780203505984

[ref80] Tajfel H. Turner J. C. (2004). “The social identity theory of intergroup behavior” in Political psychology. eds. Jost J. T. Sidanius J. (New York: Psychology Press), 276–293.

[ref81] Tan H. Lim A. (2009). Trust in coworkers and trust in organizations. J. Psychol. 143, 45–66. doi: 10.3200/JRLP.143.1.45-66, PMID: 19157072

[ref82] Tormey R. (2021). Rethinking student-teacher relationships in higher education: a multidimensional approach. High. Educ. 82, 993–1011. doi: 10.1007/s10734-021-00711-w

[ref83] Trujillo G. Tanner K. D. (2014). Considering the role of affect in learning: monitoring students’ self-efficacy, sense of belonging, and science identity. CBE Life Sci. Educ. 13, 6–15. doi: 10.1187/cbe.13-12-0241, PMID: 24591497 PMC3940464

[ref85] Uma Sankar M. Subhendu P. Bibhuti Bhusan M. (2016). Augmenting human potential at work: an investigation on the role of self-efficacy in workforce commitment and job satisfaction. Pol. J. Manag. Stud. 13, 134–144. doi: 10.17512/pjms.2016.13.1.13

[ref9003] Van Maele D. Van Houtte M. Forsyth P. (2014). Introduction: Trust as a Matter of Equity and Excellence in Education. Eds. Van Maele D. Forsyth P. VanHoutte M. Trust and School Life. Springer, Dordrecht. doi: 10.1007/978-94-017-8014-8_1

[ref86] Wang L. Byram M. (2019). International doctoral students’ experience of supervision: a case study in a Chinese university. Camb. J. Educ. 49, 255–274. doi: 10.1080/0305764X.2018.1518405

[ref87] Wang W. Mather K. Seifert R. (2018). Job insecurity, employee anxiety, and commitment: the moderating role of collective trust in management. J. Trust Res. 8, 220–237. doi: 10.1080/21515581.2018.1463229

[ref88] Woolderink M. Putnik K. van der B. H. Klabbers G. (2015). The voice of PhD candidates and PhD supervisors. A qualitative exploratory study amongst PhD candidates and supervisors to evaluate the relational aspects of PhD supervision in the Netherlands. Int. J. Doctoral Stud. 10, 217–235. doi: 10.28945/2276

[ref89] Wu C. S. (2006). An investigation of sense of identity among college students. Tennessee: University of Tennessee.

[ref9004] Xu C. Xiang F. Duan R. Miralles-Cardona C. Huo X. Xu J. (2023). An Analysis of Factors Influencing Chinese University Students’ Major Choice from the Perspective of Gender Differences. Sustainability, 15:14037. doi: 10.3390/su151814037

[ref90] Yan X. Zhu Z. Li B. Li Z. Sun Y. (2024). Roles and interaction of supervisors and students in collective academic supervision: a qualitative study in China. Beijing Int. Rev. Educ. 6, 282–309. doi: 10.1163/25902539-06030005

[ref91] Yousaf A. Sanders K. (2012). The role of job satisfaction and self-efficacy as mediating mechanisms in the employability and affective organizational commitment relationship: a case from a Pakistani university. Thunderbird Int. Bus. Rev. 54, 907–919. doi: 10.1002/tie.21511

[ref92] Zheng X. Hall R. J. Schyns B. (2019). Investigating follower felt trust from a social cognitive perspective. Eur. J. Work Organ. Psychol. 28, 873–885. doi: 10.1080/1359432X.2019.1678588

